# Are Evolution and the Intracellular Innate Immune System Key Determinants in HIV Transmission?

**DOI:** 10.3389/fimmu.2017.01246

**Published:** 2017-10-06

**Authors:** Rebecca P. Sumner, Lucy G. Thorne, Doug L. Fink, Hataf Khan, Richard S. Milne, Greg J. Towers

**Affiliations:** ^1^Division of Infection and Immunity, University College London, London, United Kingdom

**Keywords:** capsid, coevolution, HIV, innate immunity, restriction factors, sensing, transmission, Vpr

## Abstract

HIV-1 is the single most important sexually transmitted disease in humans from a global health perspective. Among human lentiviruses, HIV-1 M group has uniquely achieved pandemic levels of human-to-human transmission. The requirement to transmit between hosts likely provides the strongest selective forces on a virus, as without transmission, there can be no new infections within a host population. Our perspective is that evolution of all of the virus–host interactions, which are inherited and perpetuated from host-to-host, must be consistent with transmission. For example, CXCR4 use, which often evolves late in infection, does not favor transmission and is therefore lost when a virus transmits to a new host. Thus, transmission inevitably influences all aspects of virus biology, including interactions with the innate immune system, and dictates the biological niche in which the virus exists in the host. A viable viral niche typically does not select features that disfavor transmission. The innate immune response represents a significant selective pressure during the transmission process. In fact, all viruses must antagonize and/or evade the mechanisms of the host innate and adaptive immune systems that they encounter. We believe that viewing host–virus interactions from a transmission perspective helps us understand the mechanistic details of antiviral immunity and viral escape. This is particularly true for the innate immune system, which typically acts from the very earliest stages of the host–virus interaction, and must be bypassed to achieve successful infection. With this in mind, here we review the innate sensing of HIV, the consequent downstream signaling cascades and the viral restriction that results. The centrality of these mechanisms to host defense is illustrated by the array of countermeasures that HIV deploys to escape them, despite the coding constraint of a 10 kb genome. We consider evasion strategies in detail, in particular the role of the HIV capsid and the viral accessory proteins highlighting important unanswered questions and discussing future perspectives.

## Introduction

The primacy of transmission as a selective pressure favoring viral evasion of innate defenses is emphasized and reinforced by our understanding of the origins of HIV. The human lentiviruses HIV-1 and HIV-2 are zoonoses from simian ancestor viruses (1, 2). Antagonism of species-specific restriction factors likely determined the ability of the non-human primate viruses to cross into human hosts [reviewed in Ref. (3, 4)]. Indeed, innate effectors from both humans and non-human primates show differential patterns of restriction for simian immunodeficiency viruses (SIVs) from divergent species, as well as for HIV-1 and HIV-2 [reviewed in Ref. (5, 6)]. SIV has been transmitted from apes to humans on at least four occasions, giving rise to the M, N, O, and P groups of viruses, but the distribution and incidence of these groups vary greatly and only HIV-1 M group is pandemic (7, 8).

In the case of HIV-1, crossing a mucosal surface during sexual transmission accounts for the vast majority of new infections. However, it is not clear whether the HIV-1 ancestral viruses, in chimpanzees and gorillas, or the HIV-2 parental viruses in Sooty Mangabeys (SIVsm), are sexually transmitted diseases (STDs), and it may be that HIV-1 M has uniquely adapted to be a highly effective STD. If, as we propose, the strongest evolutionary selective forces on a virus are applied during transmission then all conserved HIV-1–host interactions must favor sexual transmission across a mucosal surface. Importantly, we consider transmission to mean the events that lead to sustained infection in the new host and not, what we imagine are frequent, cases of viral replication after exposure, which do not lead to systemic viral dissemination and peak viremia. We expect this to be the window in which the innate immune response is particularly important in protecting the host. It is our view that there is a distinction between the forces driving viral evolution within a host, for example, usage of the co-receptor CXCR4 in 50% of all hosts, that do not favor transmission and are therefore do not become fixed from host-to-host, and those that do favor transmission, and are therefore inherited. We believe that viewing HIV pathogenesis and transmission from this evolutionary perspective is essential to fully understand the antagonistic interactions between HIV-1 and the intracellular innate immune system.

Evidence for a significant genetic bottleneck during sexual HIV-1 transmission comes from the low frequency of transmission per exposure (9). Furthermore, the identification of HIV-1 founder viruses reveals that sexual transmission is established by a surprisingly low number of transmitted viral sequences (10–12). In the case of heterosexual transmission, single founder clones are typically responsible for infection, whereas several clones are usually transmitted between men who have sex with men (MSM) (13). Larger numbers are observed in intravenous transmission by injecting drug users consistent with needle use bypassing protective barriers (14). A prominent feature of acute HIV-1 infection *in vivo* is a dramatic interferon (IFN) and pro-inflammatory cytokine response (15). The sensitivity of HIV-1 to the effects of IFNs is well-established *in vitro* (16, 17). Intriguingly, characterization of transmitted founder (T/F) clones has revealed that they are less sensitive to IFN as compared with viruses isolated during the chronic phase of infection (18–22). The molecular details of the IFN-induced restriction of HIV-1 are incompletely understood, and discussed later, but an important role for the interferon-induced transmembrane protein (IFITM) family during transmission has recently been proposed (20) and is reviewed in this issue. Together, these data show how IFN and the immune response can apply powerful selective pressures during mucosal transmission.

The primary cellular targets of HIV-1 infection during transmission remain unclear. Given their high frequency in mucosa and high permissivity to infection, macrophages are likely candidates, although recent work has revealed that T/F clones are particularly poorly tropic for macrophages (23). Transmission studies of SIVmac in rhesus monkeys have suggested that inflammatory responses lead to T-cell influx and early infection of activated CD4+ T cells [reviewed in Ref. (24)]. More recent work has implicated Th17 cells as the primary target of SIVmac during vaginal inoculation (25). However, we worry that studying mucosal transmission with an unnatural virus–host pair, such as SIVmac in rhesus monkeys, in which natural sexual transmission does not occur efficiently, might be misleading. Nonetheless, the tropism of T/F sequences for CD4+ T cells is good evidence for this cell type being among the earliest targets for infection (23). Dendritic cells (DCs) and Langerhans cells (LCs), both highly abundant in mucosal surfaces, have also been implicated as primary targets during transmission (26). However, these cells are unlikely to be productively infected by HIV-1 but can capture the virus *via* uptake dependent on C-type lectins, for example, DC-SIGN and Siglec-1 (27, 28). Subsequent migration of DC to lymph nodes is thought to promote infection of CD4+ T cells by transfer of the virus, in a process called trans-infection. Despite DC not being productively infected, it is thought that these cells, particularly plasmacytoid DC (pDC), generate the high levels of systemic type 1 IFNs and pro-inflammatory cytokines in the days immediately following HIV-1 infection (15, 29–33).

**Figure 1:**
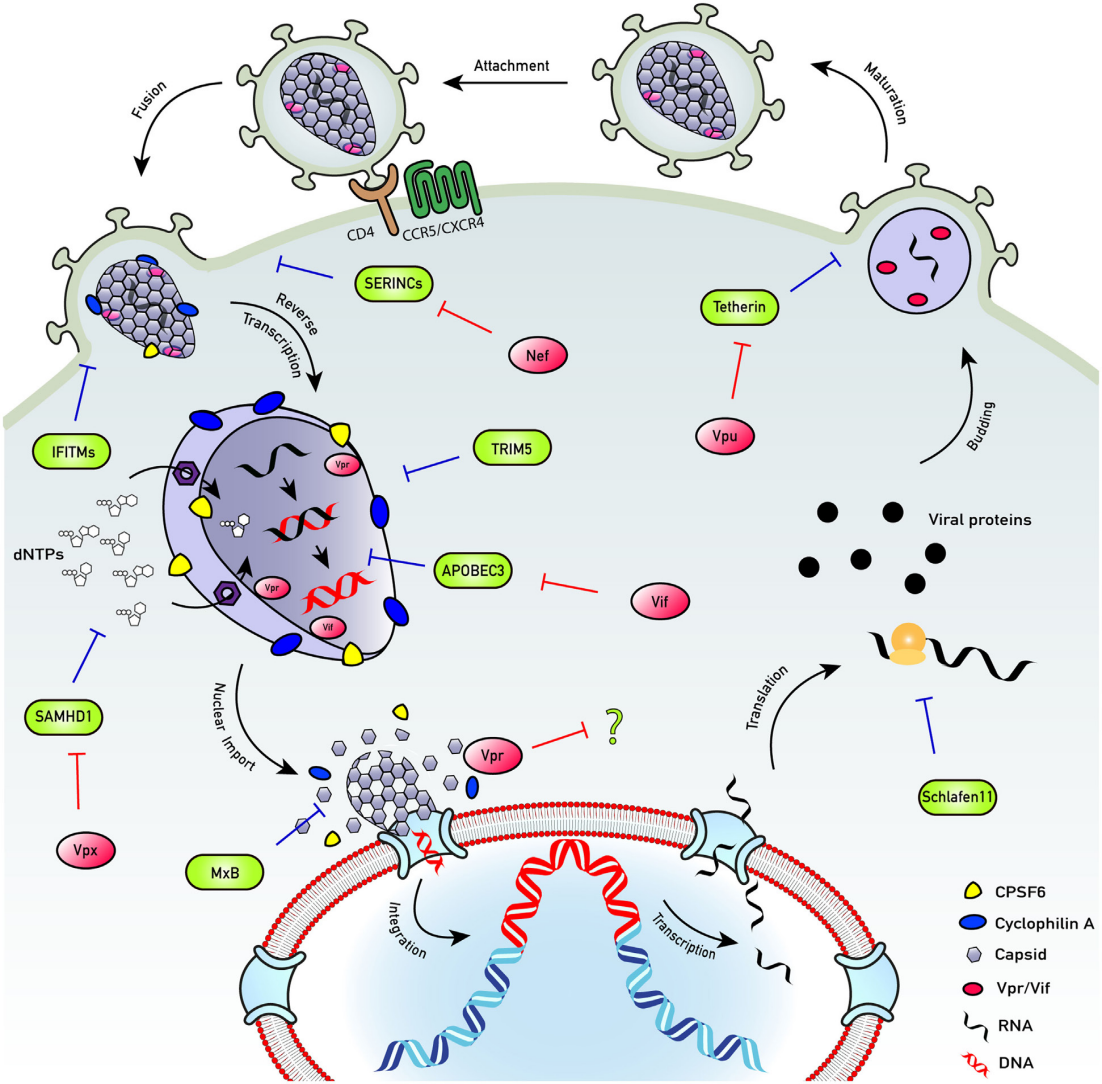
HIV life cycle. The HIV life cycle comprises a complex series of immune evasion strategies that allow successful infection of host cells and transmission between them and between individuals. To enter cells, HIV engages its envelope glycoprotein gp160 trimers with cell surface protein CD4 and co-receptor (CXCR4 or CCR5). Co-receptor usage allows conformational masking of conserved binding domains of gp120 and avoids their exposure to neutralizing antibodies. Upon fusion, capsid is released into the hostile environment of the cell where it encounters numerous innate restriction factors. However, HIV employs several mechanisms to overcome the cellular assault. While the capsid traverses the hostile cytoplasm, nucleotides are transported into the capsid cone through an electrostatic nucleotide transporter to fuel reverse transcription. Encapsidated DNA synthesis shields the viral genome from DNA sensors as well as exonucleases, e.g., TREX1. Capsid recruits cellular proteins cyclophilin A (blue) and CPSF6 (yellow), which have a role in preventing detection of the viral reverse-transcribed DNA by DNA sensors, e.g., cyclic GMP–AMP synthase (cGAS). Uncoating of successfully infectious cores may happen late, at the nuclear pore complex, or in the nucleus, in an organized manner and the viral DNA is released. The viral DNA integrates close to the edge of the nucleus to perhaps prevent activation of DNA damage responses. Once integrated, the provirus is invisible to the host cell defenses and may become transcriptionally silent, or latent. Transcription and translation of the provirus result in viral protein expression. Viral assembly occurs at the cell surface. Immature virions bud off and are released. During maturation, the protease enzyme cleaves the structural polyprotein to form mature Gag proteins, resulting in the production of new infectious virions. SERINCs: prevent fusion of viral particles with target cells. Antagonized by Nef. IFITMs: impair virus entry into target cells. Antagonized by evolving IFITM3 insensitive Env proteins. TRIM5: forms a hexagonal lattice around the capsids. Targets them for proteasomal degradation and activates innate signaling. Antagonized by evolving TRIM5 insensitive viral capsid proteins. APOBEC3: suppresses viral DNA synthesis and induces mutations in the viral DNA. Antagonized by Vif-mediated degradation. SAMHD1: restricts infection by lowering nucleotide concentrations below those, which support viral DNA synthesis. Antagonized by Vpx-mediated degradation (SIVsm/HIV-2) or infection of inactive phospho-SAMHD1 positive cells (HIV-1). MxB: restricts HIV-1 nuclear entry and possibly integration. Schlafen 11: restricts HIV-1 protein translation. Tetherin: inhibits virus release from infected cells. Antagonized by Vpu-mediated degradation.

**Figure 2:**
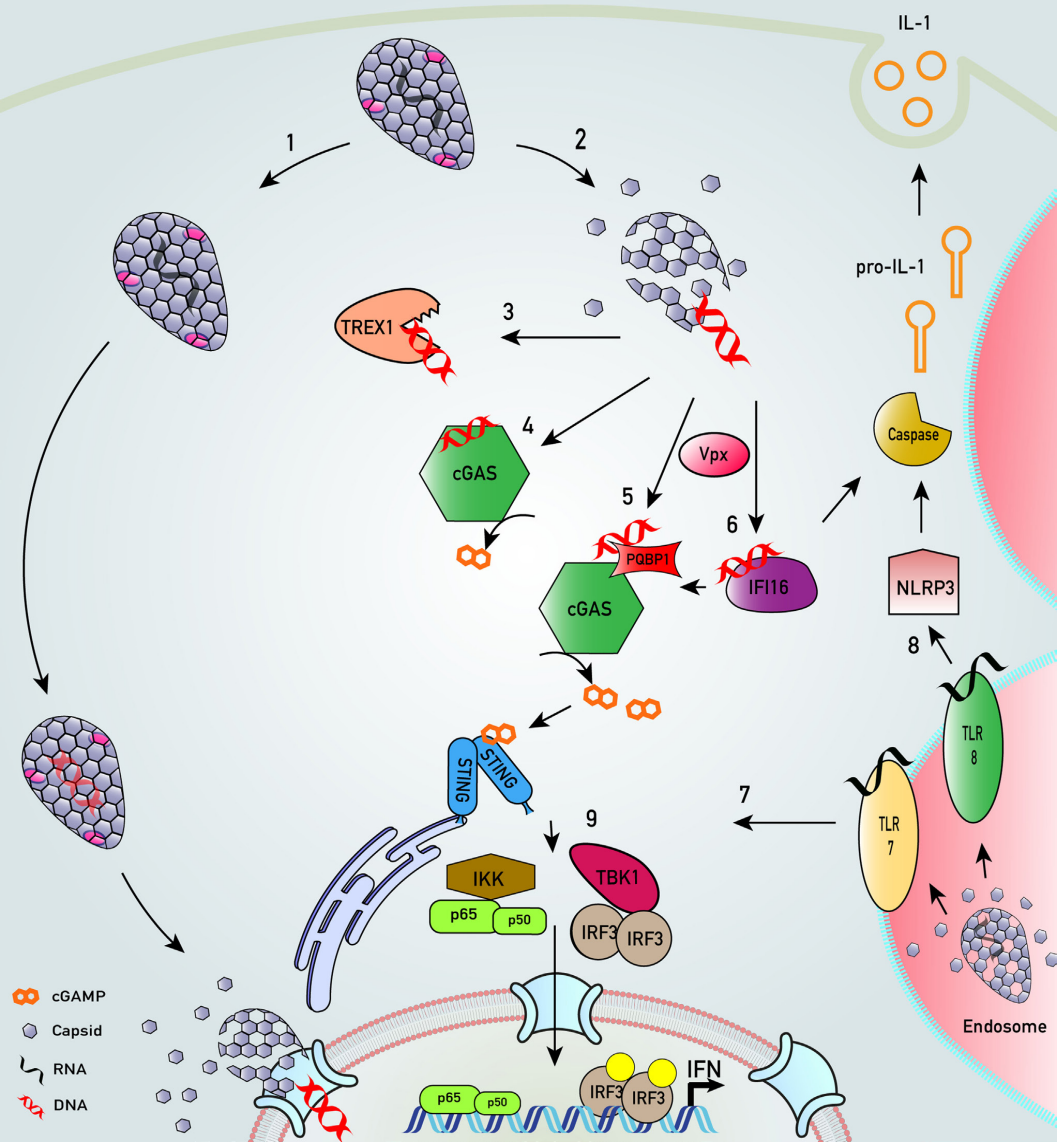
Key innate sensing pathways activated by HIV-1 particles that do not establish productive infection. (1) HIV-1 disassembly may be stochastic. Some particles remain intact, perhaps through appropriate recruitment of cofactors. We envisage encapsidated DNA synthesis and uncoating in complex with the nuclear pore complex or even in the nucleus (33, 36–40). (2) Many particles disassemble, or are disassembled, by cellular defenses that are proteasome dependent (38, 41). (3) In macrophages and T cells, cytosolic exonuclease TREX1 digests escaped HIV-1 DNA that would otherwise trigger innate DNA sensing (42). (4) In TREX1-depleted cells, escaped HIV-1 DNA is sensed by DNA sensor cyclic GMP–AMP synthase (cGAS) (42, 43). (5) In monocyte-derived dendritic cell, after SAMHD1 degradation by viral protein x (Vpx), HIV-1 DNA products are sensed by polyglutamine-binding protein 1/cGAS (44). (6) Similarly, in the presence of co-transduced Vpx, interferon-γ inducible protein 16 (IFI16) may also sense HIV-1 DNA in monocyte-derived macrophages (45). (7) HIV-1 virions in endosomal compartments of myeloid cells may not lead to productive infection but may be sensed by toll-like receptor (TLR) 7 to trigger an innate immune response that may also drive interferon (IFN) production (30). (8) HIV-1 infection of monocytic cells may also lead to TLR8-dependent assembly of NLRP3 inflammasome to activate caspase-1, which cleaves pro-interleukin-1β (IL-1β) into bioactive IL-1β (46). (9) All sensing pathways described converge on activation of transcription factors IRF3 and NF-κB that drive IFN production.

Despite the success of HIV-1 transmission, even the permissive host cell is a hostile environment for a virus. For example, the journey across the cytoplasm and into the nucleus is fraught with danger in the form of the cell-autonomous innate immune system. This intracellular immune arsenal entails a series of molecular tripwires that can mount an immediate response to invading pathogens if they are detected. Central to this defense system are pattern recognition receptors (PRRs): a diverse array of germline-encoded sensors that recognize pathogen-associated molecular patterns (PAMPs) and trigger a potent response to counteract infection, *via* activation of innate signaling pathways. This in turn induces the expression of a plethora of proteins with widespread antiviral functions that restrict infection at all stages of the viral lifecycle (Figure 1). For retroviruses such as HIV, the hazards of the cell-autonomous immune system are initially focused on the need to convert single-stranded RNA to double-stranded DNA between cell entry and integration: HIV must effectively smuggle a range of nucleic acid PAMPs past the host cell detection system. If HIV cannot negotiate these hazards it cannot replicate (Figure 2). The success story of HIV transmission therefore depends on its ability to antagonize or evade these host defenses. Every component of HIV can be defined by its individual role in the evolutionary arms race against human immunity: virus adaptation to host defenses is countered by evolution of the host cell proteins, and so on in cycles of counterevolution, described by the Red Queen Hypothesis (34), that are recorded in the genes of both organisms [reviewed in Ref. (35)].

In our view, all the host–virus interactions discussed in this review are driven by the selective forces at play during transmission. We invite the reader to consider all of the host–virus interactions we describe in the context of this perspective. Knowledge and understanding of the interactions between HIV-1 and the cell-autonomous innate immune response have rapidly expanded in recent years, and as such have been the subject of numerous reviews (47–49). Here, we provide an overview to highlight recent developments with a focus on the intracellular arms race between HIV-1 and the cell-autonomous innate immune response, from the events that determine sensing, to the downstream signaling cascades, through to the mediators of intracellular restriction, and evasion and antagonism strategies of HIV-1. For an extensive review of the extracellular interactions between HIV-1 and the innate immune system, including the IFITMs, SERINCs, and tetherin, we refer the reader to another review in this edition by the Neil group.

## Intracellular Detection of HIV by PRRs

Pattern recognition receptors fall into several families, defined either by their structure or the type of PAMP that they detect, and located in most cellular compartments including the plasma membrane, endosomes, the cytoplasm, and the nucleus (50). The PRRs implicated experimentally in the intracellular innate response against HIV are summarized in Tables 1 and 2 and Figure 2. Engagement of PRRs by PAMPs initiates a complex cascade of protein interactions leading to activation of the inhibitor of κB kinases (IKK) and the IKK-related/TBK1 kinases (51). These activate transcription factors of the NF-κB and interferon regulatory factor (IRF) families, which together coordinate the expression of antiviral type I IFNs, pro-inflammatory cytokines, and other chemokines. IFN is secreted and signals back through the IFN receptor on the surface of the infected cell and bystander cells. This causes upregulation of so-called IFN-stimulated genes (ISGs) that encode numerous proteins with direct antiviral activity (52). Importantly, a subset of ISGs is activated directly by IRFs/NF-κB allowing a more rapid activation of their expression, which is then boosted by the wave of IFN receptor-dependent signaling (53). In addition to establishing the frontline antiviral state, triggering of innate immunity is crucial for the subsequent activation of a pathogen-specific adaptive immune response. The release of pro-inflammatory mediators recruits professional antigen presenting cells to the site of infection and aids their maturation. Upon migration to the local lymph nodes, these cells then prime adaptive T and B cell responses.

**Table 1:** PRR detection of HIV in HIV target cells.

Cell type	PRR	How was the PRR implicated?	PAMP	Consequence	Reference
pDCs	TLR7	TLR7 antagonist	Purified genomic RNA	IFN, pro-inflammatory cytokines	(54)
Immature DCs	TLR8	Depletion by siRNA	ssRNA during infection	NF-κB activation, transcription of the integrated provirus	(55)
MDDC	cGAS	Depletion by shRNA, cGAMP production, and depletion by siRNA	RT products	CD86 expression, IFN and ISG induction	(32, 43, 44)
	PQBP1	Depletion by siRNA	RT products	ISG induction	(44)
	DDX3	Depletion by siRNA	Abortive RNA transcripts	IFN induction	(56)
MDM	cGAS	cGAMP production	RT products	NF-κB and IRF3 activation, IFN and ISG induction	(33, 43)
	IFI16	siRNA	RT products	Reduced replication and ISG induction	(45, 57)
	DDX3	Depletion by siRNA	Abortive RNA transcripts	IFN induction	(56)
Monocytes	NLRP3	Depletion by siRNA	Post-integration step	IL-1β and IL-18 production	(46, 58)
GECs	TLR2 and TLR4	Neutralizing Abs to TLRs	gp120	NF-κB activation and pro-inflammatory cytokine production	(59)
HLACs	IFI16	Depletion by shRNA	Abortive RT products	Pyroptosis	(60)
CD4+ T cells	DNA-PK	Chemical inhibitors	Viral integration	Cell death	(61)
	cGAS	Depletion by shRNA	Post-integration step	IFN and ISG induction	(62)
	cGAS	cGAMP production	Not determined	cGAMP production but no IFN response	(63)
	TLR7	Depletion by shRNA	Viral RNA	Anergy	(64)

*IFN, interferon; DC, dendritic cell; pDC, plasmacytoid DC; PRR, pattern recognition receptor; PAMP, pathogen-associated molecular pattern; ISG, IFN-stimulated gene; TLR, toll-like receptor; MDDC, monocyte-derived dendritic cell; MDM, monocyte-derived macrophage; RT, reverse transcription; cGAS, cyclic GMP–AMP synthase; PQBP1, polyglutamine-binding protein 1; HLACs, human lymphoid-aggregated cultures; IFI16, interferon-γ inducible protein 16; GECs, Genital epithelial cells*.

**Table 2:** PRR detection of HIV in other cell types.

Cell type	PRR	How was the PRR implicated?	PAMP	Consequence	Reference
THP-1	cGAS	Depletion by shRNA	RT products	IRF3 activation, IFN and ISG induction	(43, 57)
	IFI16	Depletion by shRNA	RT products	IRF3 activation, IFN and ISG induction	(57)
	PQBP1	siRNA and hypomorphic mutation by CRISPR	RT products	ISG induction	(44)
	NLRP3	Depletion by shRNA	Post-integration step	IL-1β production	(46)
Huh7.5	RIG-I	Cell line is defective for RIG-I	Purified secondary-structured genomic RNA	ISG induction	(65)

*IFN, interferon; PRR, pattern recognition receptor; PAMP, pathogen-associated molecular pattern; ISG, IFN-stimulated gene; RT, reverse transcription; cGAS, cyclic GMP–AMP synthase; PQBP1, polyglutamine-binding protein 1; IFI16, interferon-γ inducible protein 16*.

## Detection of HIV RNA

To date, endosomal members of the toll-like receptor (TLR) family including TLR3, TLR7, and TLR8 as well as the cytoplasmic RIG-I-like receptors (RLRs) have been described to sense RNA during infection with a range of viruses. TLR7 and TLR8 recognize ssRNA and are potent activators of NF-κB, acting *via* the signaling adaptor MyD88, whereas TLR3 recognizes dsRNA and engages the adaptor TRIF, allowing it to activate both NF-κB and IRF3. Members of the RLR family such as RIG-I and MDA-5 utilize MAVS to activate the IKK and TBK1 complexes, thus activating both the NF-κB and IRF3 arms of innate signaling [reviewed in Ref. (66)] (Figure 2).

Most studies implicating RNA sensing in the detection of HIV-1 have been based on transfection of either purified full-length HIV RNA or genome-derived oligos (Table 1). Evidence for whether these sensors are engaged during viral infection of target cells is lacking. In our view, a significant limitation of transfection-based sensing experiments is that they deliver naked RNA or genome-derived oligos directly into host cells, whereas during infection the virus uses complex evasion strategies, including wrapping the genomic RNA tightly into complexes with the nucleocapsid and other viral replicase proteins, and/or delivering it into the cell in intact protective viral capsids. Depending on the transfection method, the RNA may also be delivered to cellular compartments where it would not normally encounter sensors during infection. It remains unclear whether HIV-1 genomic RNA is accessible and can be sensed in the cytoplasm during infection. One study has indicated this is possible in monocyte-derived dendritic cells (MDDCs) and monocyte-derived macrophages (MDMs), as the RNA helicase DDX3 was able to detect abortive HIV-1 RNAs, which induced DC maturation and type I IFN responses dependent on the adaptor MAVS (56).

Using transfection methods, HIV RNA has been reported to be detected by members of both the TLR and RLR families. Guanosine- and uridine-rich ssRNA oligonucleotides derived from the HIV-1 LTR were found to stimulate both pDCs and macrophages to secrete IFNα and pro-inflammatory cytokines such as TNFα (67). Using murine cells deficient for various TLRs as well as TLR overexpression in 293T cells, the authors concluded that TLR7 and TLR8 were responsible for the sensing of HIV-1-derived ssRNA (67). TLR7 antagonists have been shown to inhibit cytokine release by pDC incubated with purified HIV-1 RNA (54).

The cytoplasmic RLRs have also been implicated in the detection of HIV-1 RNA through transfection-based studies. Secondary-structured genomic RNA induced ISG expression in peripheral blood mononuclear cells (PBMCs), independent of endosomal TLR signaling (65). MAVS and RIG-I were implicated in this study using murine bone-marrow derived macrophages deficient for MAVS, and Huh7.5 cells with defective RIG-I function. Purified monomeric and dimeric forms of HIV-1 genomic RNA were further shown to be potent PAMPs and inducer of ISGs in PMA-differentiated THP-1 cells (68). Using deficient MEFs, detection of this genomic RNA was shown to be RIG-I- but not MDA-5-dependent, although detection of HIV-1 RNA by these sensors was not demonstrated in human cells.

## Detection of HIV Reverse Transcription (RT) Products

A recently discovered and rapidly expanding arm of innate immunity research is the detection of viral DNA by cytoplasmic DNA sensors. Our knowledge of cytoplasmic DNA sensing has lagged behind that of RNA sensing, perhaps because, while a large proportion of tissue culture adapted cell lines are competent for sensing *via* RLRs and TLRs, the DNA-sensing pathways are generally defective in cell lines. For 293T and HeLa cells, some of the most transfectable cell lines, this has been attributed to expression of the viral oncoproteins E1A and E7, respectively, involved in transformation of the cell lines, which bind and inhibit STING, a central component of DNA signaling pathways, Figure 2 (69). Indeed, transfectability may be dependent on defective DNA sensing. As a result, the use of primary cells, and the few cancer cell lines that are competent for DNA sensing (e.g., monocyte-like THP-1 cells), has been crucial to the expansion of our knowledge in this area. While cells such as THP-1s respond to a range of innate immune PAMPS and agonists, it remains unclear, even for these cells, whether they are as responsive to stimulation as primary macrophages.

Most DNA sensors that have been described to date utilize the ER resident signaling protein STING to activate NF-κB and IRF3 (70), Figure 2. STING is a direct sensor of cyclic dinucleotides (71), the best characterized of which, 2′–3′ cGAMP, is synthesized by the sensor cyclic GMP–AMP synthase (cGAS) upon binding to DNA in the cytoplasm (72–74). Upon engaging cGAMP, STING translocates *via* the Golgi to distinct perinuclear regions where it can activate the IKK and TBK1 complexes and thus drive a type I IFN response (75).

Some of the earliest evidence that HIV-1 produces a stimulatory DNA PAMP during infection was obtained in human CD4+ T cells and macrophages that had been depleted for the cytosolic exonuclease TREX1 (42). In this study TREX1 was suggested to digest unencapsidated HIV-1 DNA that would otherwise activate a type I IFN response in a STING-dependent manner. The sensor responsible for the detection of HIV DNA was later described by multiple groups to be cGAS (32, 33, 43, 57). Two groups measured cGAS- and STING-dependent ISG responses in monocyte-like THP-1 cells infected with VSV-G-pseudotyped HIV-1 vector, which were dependent on RT but independent of integration (43, 57). Gao et al. were able to measure cGAMP production in primary MDMs and MDDCs infected with HIV-1 in the presence of SIV virus-like particles (VLPs). SIV VLPs were used to deliver SIV accessory protein viral protein x (Vpx) to inhibit the restriction factor SAMHD1, thereby allowing HIV-1 infection. cGAS-dependent sensing of HIV-1 and HIV-2 has also been implicated in MDDCs pretreated with Vpx (32).

The function of cGAMP as a second messenger goes beyond the infected cell, as it can also pass through gap junctions and activate an antiviral response in neighboring cells in a STING dependent, but cGAS-independent manner (76). cGAMP can also be packaged in lentiviral virions themselves and is spread in this way to neighboring cells with infection (77, 78).

Interferon-γ inducible protein 16 (IFI16), a member of the PYHIN family, was originally described as a STING-dependent DNA sensor for transfected DNA and herpes simplex virus-1 (79). However, this sensor may be capable of detecting both single- and double-stranded HIV-1-derived DNA in THP-1 cells and primary MDMs (57). Depletion of IFI16 by siRNA in primary MDM led to enhanced replication of HIV-1, implicating this protein in the innate detection of HIV, although IFN or ISG induction in these cells was not measured in this study (57). Reduced ISG induction in IFI16-depleted primary MDM infected with HIV-1 BaL in the presence of SIV VLPs was, however, demonstrated in a follow-up study (45).

Polyglutamine-binding protein 1 (PQBP1) was recently identified in a targeted RNAi screen in MDDC and described as a DNA sensor that directly bound to reverse-transcribed HIV-1 DNA and interacted with cGAS to activate an ISG response (44). A role for this protein was also demonstrated in THP-1 cells, as silencing of PQBP1 led to reduced innate immune activation induced by HIV-1 VSV-G pseudotyped vector. In these experiments, co-infection with SIVmac VLPs antagonized SAMHD1. In both cell types, the authors measured a significant reduction in cGAMP production upon infection after PQBP1 depletion, leading them to conclude that it was required for an optimal cGAS/STING response to HIV-1 DNA in myeloid cells (44). A similar proximal role in augmenting the cGAS/STING pathway has now also been suggested for IFI16. In THP-1 cells, IFI16 enhanced cGAMP production upon DNA stimulation and aided the recruitment of TBK1 to STING to enhance IRF3 activation (45). Furthermore, IFI16 enhanced STING activation and signaling complex formation in keratinocytes, although in this study the authors did not find a role for IFI16 in cGAMP production, suggesting that cell type-specific roles for this protein may exist (80). These recent studies suggest that cGAS and STING constitute a central pathway that senses HIV-1 DNA in the cytoplasm, with proteins including PQBP1 and IFI16 somehow enhancing this signaling rather than acting independently as DNA sensors themselves (Figure 2).

An outstanding question in the field is which HIV-1 RT products are the major PAMP during infection. During RT, both single-stranded and double-stranded DNA are generated, as well as RNA:DNA hybrids. While both forms of DNA were recognized in an IFI16-dependent manner when transfected into THP-1 cells, Jakobsen and colleagues were not able to measure a significant innate response to RNA:DNA hybrids (57). By contrast, transfection of murine DCs and human PBMCs with RNA:DNA hybrids induced robust IFN and pro-inflammatory cytokine release, which was dependent on TLR9 (81). cGAS has also been implicated in the detection of RNA:DNA hybrids in PBMCs and PMA-differentiated THP-1 cells (82). Whether these transfection experiments reproduce the PAMP production and exposure seen during infection, and whether these sensors detect RT-derived hybrids during infection of relevant primary human target cells, remains to be determined.

## Detection of Non-Nucleic Acid Components of HIV

In addition to the detection of nucleic acids, some studies have suggested HIV proteins may act as PAMPs. The E3 ubiquitin ligase tripartite motif protein 5 (TRIM5α), is a capsid-binding restriction factor. A seminal study by Pertel et al. proposed that this protein also functions as a PRR to induce innate signaling upon recruitment of retroviral capsids (described in more detail below) (83). The restriction factor tetherin, which prevents newly synthesized virions from budding from the infected cell, has also been reported to act as a PRR that activates innate immune signaling cascades (84, 85), this is reviewed elsewhere in this edition.

## Activation of Inflammasomes by HIV

Inflammasomes are multiprotein complexes, found in myeloid cells and T cells, and activated by a wide variety of PAMPs and host-derived danger-associated molecular patterns. The innate sensors capable of activating inflammasomes include members of the NOD-like receptor family, the RLRs, and the DNA-sensing-associated proteins AIM2 and IFI16. Engagement of these receptors leads to the formation of a platform for caspase-1 activation and subsequent proteolytic maturation and secretion of the pro-inflammatory cytokines such as interleukin-1β (IL-1β) and IL-18, or induction of pyroptosis, an inflammatory form of programmed cell death [reviewed in Ref. (86)].

Two studies to date have described inflammasome activation in monocytic cells by HIV-1 (46, 58). IL-18 production by monocytes exposed to HIV-1 was dependent on endocytosis rather than infection, and both studies found that TLR-8 activation was required for induction of *pro-IL-1*β whereas cleavage into its active form and release was dependent upon NLRP3 and the inflammasome adaptor protein ASC (46, 58). Guo et al. further demonstrated that inflammasome activation occurred post-integration leading them to suggest HIV transcripts as potential PAMPs.

## Sensing of HIV DNA in CD4+ T Cells

In contrast to the classical ISG and pro-inflammatory cytokine response observed in HIV-infected cells of myeloid origin, innate immune sensing in CD4+ T cells has been described to lead to cell death. It is clear that HIV-1 replication in CD4+ T cells leads to massive cell death, but there are conflicting reports regarding the role of innate immune sensing and the mechanisms of this process (61, 87). One possible explanation for these discrepancies relates to the origin and activation status of the T cells used in each study.

Studies from the Greene lab using human lymphoid-aggregated cultures (HLACs) from tonsillar tissue showed that abortive HIV-1 infection of these cultures led to significant cell death, which did not require viral integration (88). This study suggested that in many of the T cells in an infected culture, viral DNA synthesis occurred, but that the infection arrested before integration. The authors proposed that sensing of HIV-1 DNA in these abortively infected cells was responsible for the T cell death that drives T cell loss and eventually AIDS. A follow-up study demonstrated that death by apoptosis occurred only in the small percentage of cells in the culture that were productively infected and that the vast majority of cells died *via* caspase-1-mediated pyroptosis after abortive infection (87). They reported that IFI16 was the DNA sensor responsible for detecting the incomplete RT products in the abortively infected T cells (60). This group subsequently demonstrated that, in contrast to CD4+ T cells in HLACs, PBMC-derived CD4+ T cells are resistant to death by pyroptosis (89). They attributed this to the resting status of peripheral blood-derived CD4+ T cells, which could be overcome by coculture with lymphoid-derived cells, resulting in pyroptosis on HIV-1 infection (89). In contrast to these studies, the Nabel lab used PBMC-derived primary CD4+ T cells and found that, in these cells, HIV-1 induced cell death was associated with productive HIV-1 infection and dependent on integration (61). Cell death was accompanied by DNA-PK activity and phosphorylation of p53 and H2AX. The authors proposed that HIV-1 integration was detected by the DNA repair enzyme and DNA sensor, DNA-PK, as chemical inhibition of DNA-PK prevented cell death.

Interestingly, more recent publications are now beginning to address whether CD4+ T cells can in fact sense HIV-1 RT products in a manner more similar to myeloid cells. Again there are conflicting reports, with some studies measuring a type I IFN response after HIV-1 infection of T cells (60, 62, 88), while others have been unable to detect such a response (63, 90). Vermeire and colleagues observed an IFN and ISG response in PHA/IL-2-activated primary CD4+ T cells that was cGAS dependent and required provirus integration (62). In another study, cGAMP production was also detected in CD4+ T cells but in this case cGAMP did not lead to IFN production by the infected cells (63). Interestingly, the authors found that the cGAMP from the infected T cells could be transferred and activate a STING-dependent ISG response in macrophages through Env-induced membrane fusion sites, identifying an alternative mechanism by which T cell infection can contribute to local IFN production *via* macrophages.

## Intracellular Restriction of HIV

Interplay between cellular restriction factors and HIV-1 occurs at every stage of its lifecycle and the virus uses a combination of evasion and antagonism strategies to achieve infection and replication (Figure 1). Our advancing understanding of the mechanisms of viral replication and innate immunity mean that any strict criterion for defining restriction factors rapidly becomes outmoded. It would be a shame for poorly thought out nomenclature to constrain creative thinking and understanding of innate immunity. We take the view that any protein with well-characterized antiviral activity can be considered a restriction or resistance factor. A current exciting research focus is the intersection between traditional direct-acting restriction factors and innate immune signaling. An emerging and important feature of restriction factors is to act a sensor for the presence of infection, as has been demonstrated for TRIM5, tetherin, and TRIM21 (83, 84, 91).

## TRIM5α

TRIM5α targets incoming retroviral capsids soon after they enter the host cell to block infection before integration (Figure 1). It belongs to the large TRIpartite Motif (TRIM) family of proteins, encoded by over 100 genes in humans, which are involved in diverse cellular processes. Many TRIM proteins, including TRIM5α, are upregulated by type I and II IFNs and have direct antiviral and antimicrobial roles, in addition to less well defined regulatory roles in innate immunity in general [reviewed by Rajsbaum et al. (92)]. Among the TRIM family members, the TRIM5 locus exhibits the greatest rate of positive selection across primate genomes, probably due to selective pressure from direct interactions with retroviruses (93). TRIM5α has been extensively reviewed since it was discovered in 2004 (94, 95); however, recent reports have extended ideas on restriction specificity and have shed significant light on its antiviral mechanism and the role of ubiquitin in this process (96).

TRIM5α represents an important barrier to zoonotic retroviral transmission. It was first identified as an important contributor to the innate resistance of Old World Monkeys to HIV-1 infection, targeting incoming viral capsids to prevent RT (97). Further study of TRIM5 antiviral specificity revealed that each primate TRIM5 restricts a different subset of lentiviruses (97, 98). The importance of TRIM5α for species-specific restriction of HIV-1 is illustrated by the observation that the only monkeys permissive for SIV/HIV-1 chimeras bearing HIV-1 capsid are pigtailed macaques that are homozygous for a TRIMCyp protein that cannot restrict HIV-1 (99, 100).

The defining tripartite domain architecture of the TRIM family, comprises an N-terminal RING domain with E3 ubiquitin ligase activity, a B-box domain and a coiled-coil region that both mediate multimerization through protein–protein interactions (83, 101). TRIMs have various domain types at the C-terminus and, like many TRIMs, TRIM5α has a C-terminal PRYSPRY, also called a B30.2 domain, which is not present in splice variants that lack retroviral restriction activity. Restriction specificity is dependent on direct interaction between the viral capsid protein and the TRIM5α PRYSPRY (102–104). In an intriguing evolutionary arms race, TRIM5α has been modified independently in several simian species by swapping the PRYSPRY for a lentivirus-targeting cyclophilin A (CypA)-like domain. This is derived from retrotransposition of a CypA cDNA (99, 105–110). These observations indicate the importance of CypA to the virus and the plasticity of TRIM5α antiviral evolution.

Until recently, human TRIM5α has been thought to have poor restriction activity against HIV-1. This has been explained by a lack of interaction between the human TRIM5α PRYSPRY and the HIV-1 capsid. Indeed, human TRIM5α can be modified to restrict HIV-1 by a single point mutation in the PRYSPRY (104) or by replacing the whole domain with the rhesus macaque TRIM5α PRYSRY (102). Certain primary isolates of HIV-1 have been found to be more sensitive to TRIM5α than lab strains. Indeed, it has been suggested that T cell escape mutations in the capsid target of TRIM5α may drive HIV-1 to be TRIM5α sensitive (111). Likewise, TRIM5α polymorphisms and expression levels have been associated with differential rates of HIV-1 acquisition and disease progression, supporting a role for human TRIM5α in HIV-1 transmission and pathogenesis *in vivo* (112, 113). Whether these observations are explained by direct TRIM5α restriction of HIV-1, or by its role in innate immune signaling (83) remain unclear.

A landmark study from the Geijtenbeek lab recently demonstrated that human TRIM5α contributes to restriction of HIV-1 in a cell type, and entry pathway, specific manner (114). LCs, resident in mucosal surfaces, are innately resistant to HIV-1 due to their unique C-type lectin receptor langerin, which mediates uptake of HIV-1 but directs virus into Birbeck granules for degradation (115). By investigating the specific mechanism of langerin-dependent restriction, Ribeiro et al. discovered a role for TRIM5α (114). Depletion of TRIM5α in primary LCs, or a Langerhans-like cell line (MUTZ-LCs), resulted in increased infection and enhanced transmission to cocultured CD4+ T cells. Critically, expression of langerin in a cell line (U87) allowed endogenous TRIM5α to restrict HIV-1, but only when langerin, and not the VSV-G receptor, was used as the virus entry receptor. Association of langerin and TRIM5α in cells was suggested by co-immunoprecipitation. This receptor mediated targeting to TRIM5α-dependent restriction was specific to langerin and was not observed when HIV-1 entered MDDC *via* the C-type lectin receptor DC-SIGN, or, of course, T cells *via* CD4 (114).

The study went on to show a role for autophagy in human TRIM5α-mediated restriction of HIV-1. TRIM5α was associated with components of the autophagy machinery in steady state conditions by co-immunoprecipitation and restriction led to an increase in autophagosome formation. Silencing autophagy proteins Atg16LI and Atg5 ablated the langerin-dependent TRIM5α-mediated restriction of HIV-1 (114). In our view, these data do not conflict with previous reports demonstrating receptor independent, PRYSPRY dependent, interaction between TRIM5α and capsid to define antiviral specificity. Rather, they provide evidence for a role for TRIM5α in restriction of HIV-1 by autophagy when langerin is utilized as an entry receptor. We expect that as the details of this restriction mechanism are uncovered the differences between this autophagy dependent, and previously described proteasome dependent, mechanisms will be clarified and a novel role for TRIM5α in autophagy defined.

Structural studies have shed significant light on the classical antiviral mechanism of TRIM5α. There is evidence that TRIM5α forms hexagonal assemblies on the surface of retroviral capsids, mimicking the organization of the hexameric capsomeres (116). Hexagonal lattice formation may position multiple C-terminal PRYSPRY domains, which interact with the capsid with low affinity and specificity, so as to promote binding through avidity effects. This observation was recently recapitulated using electron microscopy with recombinant full-length rhesus macaque TRIM5α proteins and purified native intact HIV-1 capsid cores. The B-box 2 domain appears responsible for mediating TRIM5α–TRIM5α interactions that drive higher order assembly of TRIM5α into multimers and are essential for restriction activity. The hexagonal TRIM5α nets are thought to have conformational flexibility enabling them to form on divergent retroviral capsid sequences, with different capsomere curvature and conformation. This model could explain the broad recognition of divergent viruses associated with TRIM5α antiviral activity (116, 117).

Formation of TRIM5α complexes on an incoming virion is reported to promote rapid capsid disassembly and premature uncoating (118). However, it is clear that the process of viral disassembly and disruption of viral DNA synthesis is dependent on ubiquitin-dependent recruitment of the proteasome (96, 119). Indeed, the fact that preventing proteasomal degradation of the TRIM5α–virus complex restored restricted viral DNA synthesis was the first hint that viral DNA synthesis occurs inside an intact capsid, a model that is gaining increasing traction (40). HIV-1 capsid uncoating is normally a highly regulated process and so premature uncoating by TRIM5α/proteasomes likely accounts for the observed block to RT.

A consequence of TRIM5α-capsid binding is activation of its RING domain E3 ubiquitin ligase activity (83). This results in complex TRIM5α autoubiquitination and enhanced proteasomal turnover, suggesting that TRIM5α targets capsids for proteasomal degradation (96, 120). Recent mapping of sequential TRIM5α autoubiquitination steps using a combination of biochemical and genetic approaches has implicated a series of E2 conjugation enzyme and ubiquitin linkages. Ube2W first attaches single Ub molecules to TRIM5α, which are then extended into polyUb chains through Lys63-linkages catalyzed by the heterodimeric E2 enzyme complex Ube2N/Ube2V2. Each of these steps was required for human TRIM5α restriction of murine leukemia virus (MLV) RT (96).

TRIM5α appears to serve as capsid PRR activating transcription factors NF-κB and AP-1 and resulting in pro-inflammatory cytokine synthesis that could contribute to the antiviral state and modulate adaptive responses (83). Inhibitors of TAK1 signaling, or depletion of pathway components, rescues some degree of TRIM5α restricted infections in myeloid cells suggesting this is a component of TRIM5α activity. A recent study further correlated the ability to induce signaling with retroviral restriction activity, although this was demonstrated using murine TRIM5α orthologs modified to be able to target HIV-1 capsids (121). It will be interesting to test whether activation of NF-κB and AP-1 pathways occurs after human TRIM5α inhibition of HIV-1 in LCs, and whether this contributes to restriction in this cell type.

Finally, it has recently been proposed that as well as amplifying the innate immune response, TRIM5α directly enhances the potency of CD8+ T cell responses to infected cells. Rhesus macaque TRIM5α restriction of HIV-1 boosted HIV-1 specific T cell activation and inhibition of infected cells *in vitro*. It is possible that TRIM5α mediated recruitment of virus to proteasomes may lead to increased peptide availability for MHC presentation (122).

## SAMHD1

Sterile alpha motif and histidine–aspartate domain containing protein 1 (SAMHD1) was identified as the restriction factor targeted by the SIV accessory protein Vpx in myeloid cells (123, 124). Shortly after this SAMHD1 was found to be a deoxynucleoside triphosphate triphosphohydrolase (dNTPase) that restricts infection by lowering nucleotide concentrations below those which support viral DNA synthesis (125). In the case of viruses such as SIVsm and HIV-2, Vpx directs proteasomal degradation of SAMHD1 by recruitment of the host cell cullin-4 ligase substrate receptor DDB1- and CUL4-associated factor 1, DCAF1, also known as Vpr-binding protein, for polyubiquitination (123). In this way, Vpx provided either packaged into VLPs for co-transduction or stably expressed in cell lines, is able to counteract SAMHD1 restriction of HIV-1 infection.

SAMHD1 comprises three main regions: the N-terminus, a catalytic core HD domain, and the C-terminus. Most reports attribute HIV-1 restriction to the dNTPase activity of the HD domain, which inhibits viral DNA synthesis by reducing the dNTP supply for RT (126). Mutations of key residues in the HD region cause SAMHD1 to lose its ability to restrict HIV-1 (124). Depletion of SAMHD1, using siRNA or by delivering SIV Vpx in *trans*, boosts both intracellular dNTP pools and HIV-1 replication. Indeed, SIV VLPs have regularly been used as a tool to deplete SAMHD1 thereby allowing the study of antiviral properties that would otherwise be masked by SAMHD1 activity. HIV-1 reverse transcriptase mutants with reduced dNTP affinity are consistently more sensitive to SAMHD1 restriction (127). Some studies have proposed additional antiviral activities for SAMHD1. For example, Ryoo et al. showed that overexpression of RNAse-active but dNTPase-inactive SAMHD1 mutants, identified through biochemical assays, are able to restrict HIV-1 (128). They also observed modest increases in HIV-1 RNA stability following transient SAMHD1 depletion. Other groups have suggested that RNase activity may be an artifact of contaminated samples (129, 130). Certainly, the SAMHD1 structural work is consistent with its role as a dNTPase (125).

SAMHD1 is widely expressed in diverse human tissues but *in vitro* appears to only restrict HIV-1 infection in non-dividing cells, perhaps because they typically have low nucleotide levels within the range of SAMHD1 control. Conversely, most rapidly dividing cell lines have high nucleotide levels that may exceed the inhibitory capacity of SAMHD1.

Unlike other restriction factors, where expression alone is typically sufficient to block infection, SAMHD1 antiviral activity is often not measurable in dividing cell lines. This may be because it is regulated by cell cycle-dependent phosphorylation as well as dNTP levels. SAMHD1 is inactivated by cyclin-dependent kinase (CDK)-mediated phosphorylation at C-terminal residue T592 (131). Structural studies have associated this inactivation with unstable tetramer structure and increased dissociation to catalytically inactive monomers and dimers (127, 129). The local dNTP environment also regulates SAMHD1 structure and function. Binding of dNTPs to the C-terminal allosteric regulation domains is required to activate tetramerisation and optimal catalytic activity (125, 132–134). SAMHD1 mutants that are unable to oligomerize are unable to restrict HIV-1 and this correlates with their inability to reduce dNTP pools (127, 133). One model to explain SAMHD1 activity in non-dividing cells is that the absence of CDK-mediated phosphorylation means that the small available dNTP pool is directed toward the C-terminal allosteric sites (127), leading to durable tetramer formation, dNTPase activity and HIV-1 restriction.

Recent work has revealed a crucial role for the CDK-mediated regulation of SAMHD1 in determining permissivity of myeloid cells to HIV-1 infection. Mlcochova et al. showed that T592 phosphorylation and thus SAMHD1 antiviral activity are dynamic in primary human MDM (135). They propose that macrophages, and likely other myeloid cells, exist in two states through which all of the cells periodically cycle. The first, a typical G0 state, characterized by active dephosphorylated SAMHD1, lack of the cell-cycle marker minichromosome maintenance complex component 2 (MCM2) and resistance to HIV-1; and the second, described as a G1-like state, permissive to HIV-1 and characterized by expression of MCM2 and inactive phosphorylated SAMHD1. Critically, though SAMHD1 phosphorylation in this model is CDK1 dependent and linked to MCM2 expression, both states exist without measurable DNA synthesis or cell division (135). These observations provide a plausible explanation for the ability of HIV-1 to infect myeloid cells despite the apparent presence of active SAMHD1 within the cell population. They may also explain the lack of an HIV-1 encoded SAMHD1 antagonist, though the question of why other viruses may have evolved one in Vpx remains open.

Inhibition of SAMHD1 restriction activity by phosphorylation is widely accepted, but some studies in non-permissive differentiated U937 cells or using biochemical assays have suggested that dephosphorylation does not affect dNTPase activity (136, 137). We note that the technical challenges of measuring intracellular dNTP levels, and, more particularly, direct enzyme activity across cell populations with unsynchronized cell-cycle status are consistently highlighted in the literature (127, 130).

There is consensus that SAMHD1 binds single-stranded nucleic acids (129, 138). However, whether there is specificity for this interaction remains unclear. In macrophages, HIV-1 RNA co-immunoprecipitates with SAMHD1 (128) and in biochemical assays ssRNA binds monomeric and dimeric SAMHD1 to inhibit oligomerization and dNTPase activity (130). This has not been recapitulated in cells but leaves open the possibility that binding of SAMHD1 to nucleic acids may represent a further restriction mechanism.

A number of SAMHD1 mutations are implicated in some cases of Aicardi–Goutieres syndrome, a condition characterized by elevated systemic IFN levels, dependent on innate sensing of endogenous retroviruses (139). This has been attributed to loss of SAMHD1-mediated restriction of LINE-1 (long interspersed element-1) retrotransposition that generates a DNA PAMP (140). Intriguingly, restriction of endogenous retroviruses was not sensitive to Vpx and was retained in the presence of a SAMHD1 catalytic site mutant, leading some to propose that SAMHD1 may sequester ssRNA to prevent sensing during homeostasis (141).

Unlike other restriction factors, SAMHD1 expression is not induced by type I IFN in human DCs, macrophages or CD4+ T cells (142, 143), the main target cells of HIV-1. SAMHD1 induction has been reported in HEK 293T and HeLa cells (144). It is possible that SAMHD1 is activated in response to type I IFNs, which have been shown to reduce phosphorylation at residue T592 in MDMs and MDDCs, which would promote SAMHD1 tetramerization and catalytic activation (131). Together, these data implicate SAMHD1 as a component of a typical IFN-inducible antiviral response.

Lentiviral accessory proteins are often not conserved in their functions. For example, tetherin is antagonized by HIV-1 Vpu but the parental virus SIVcpz uses nef for this purpose. In a similar way, several viruses use Vpr, rather than Vpx, to antagonize SAMHD1. SAMHD1-degrading Vpr proteins are encoded by SIV syk (SIV that infects Sykes’ monkey), SIV deb (De Brazza’s monkey), and SIV agm (African green monkey) lineages (145). It is tempting to suggest that HIV-1 has gained advantage from avoiding a SAMHD1 degradation phenotype. Some have proposed that HIV-1 transmission *in vivo* is enhanced by avoiding sensing and activation of antiviral intracellular innate responses in dendritic and myeloid cells, perhaps evidenced by fewer cases of Vpx encoding HIV-2 than HIV-1 (146, 147). The model is that abrogation of SAMHD1 leads to HIV-2 DNA synthesis, which can then activate innate immune DNA sensing, particularly in DC. Consistent with this theory, HIV-1 infection of MDDC and MDM only results in cGAMP production when SAMHD1 is inhibited by pretreatment with VLPs containing Vpx (43, 148). Further, chronic HIV-2 infection is often characterized by stable CD4+ T cell counts, which may reflect an inability to efficiently establish high levels of infection in these cells *in vivo* (149). However, there are likely to be many differences between HIV-1 and HIV-2 that lead to the lower pathogenicity and transmission rates of HIV-2 as compared with HIV-1 and the role of Vpx in these differences remains poorly defined. Furthermore, there is as yet no good evidence that Vpx enhances replication in myeloid cells *in vivo*.

## APOBEC3

Apolipoprotein B mRNA-editing, enzyme-catalytic, polypeptide-like 3 proteins (APOBEC3 or A3) belong to the family of single-stranded DNA deaminases. A3s are IFN-inducible and restrict HIV-1 primarily by suppressing viral DNA synthesis and inducing mutations in the viral DNA leading to replication incompetent proviruses (148, 150–152). Seven A3 enzymes have been identified: A3A A3B, A3D, A3F, A3G, and A3H are all active against HIV-1, and A3C may be inactive (4, 153). A3G is the most well defined anti-HIV APOBEC3 protein and was the first to be described to have a role in innate immunity through its ability to block HIV-1 replication (150). It is expressed in CD4+ T cells and MDM (154). The importance of APOBEC3 proteins in transmission and species-specific replication of HIV-1 is underlined by the observation that HIV-1 can be made to replicate in pigtailed macaques by changing only the APOBEC3-antagonizing HIV-1 accessory gene Vif (100).

To restrict HIV-1, A3 proteins must be packaged into viral particles and access the viral DNA in the infected cell (155). For example, A3A is not packaged but can be made to restrict HIV-1 by forcing incorporation into virions by fusing it to the packaged viral accessory protein Vpr (156). A3G is packaged into virus particles through its interaction with cellular or viral RNAs bound to the nucleocapsid domain of the Gag polyprotein (157). In the absence of the antagonistic viral accessory protein Vif (described below), A3G suppresses DNA synthesis and catalyzes the deamination of cytosines to form uracils in the minus strand of the reverse-transcribed single-stranded DNA, resulting in G to A mutations in the plus strand of the viral DNA (158, 159). The hypermutated proviral DNA that results is defective and unable to produce infectious progeny (160).

A3G disruption of HIV-1 DNA synthesis occurs at several steps. A3G prevents tRNA binding to the primer binding site in the viral RNA (161), minus and plus strand transfer (162), and primer tRNA processing and DNA elongation (152, 163). The studies reporting lack of HIV-1 restriction by the deaminase inactive A3G mutant (E259Q) should be considered in light of reports that show that the A3G E259Q mutant is also defective for RNA binding and therefore unable to inhibit HIV-1 DNA synthesis to the same extent as the wild-type A3G (164).

## SUN2

SUN2 (also known as *UNC84B)* was originally identified as a potential innate immune effector with specific antiretroviral activity in an overexpression screen for ISGs against a range of different viruses (165). SUN2 is an integral membrane protein that spans the inner nuclear membrane and forms part of a multiprotein complex (LINC) that physically bridges the nucleoskeleton and cytoplasm (166). Several recent studies published in quick succession have suggested that manipulation of SUN2 can either inhibit or promote HIV-1 infection, depending on the level of expression (167–169).

Studies have found that SUN2 is constitutively expressed in human cells and is in fact not upregulated by IFNs (167–170). Several groups have confirmed that SUN2 overexpression leads to a block to HIV-1 infection and replication, as originally reported in Ref. (165). However, endogenous levels of SUN2 did not have antiviral activity. This suggests that SUN2 overexpression has antiviral activity through a dominant negative effect rather than through having specific innate antiviral properties.

SUN2 was included in the original ISG screen based on microarray data from primary chimpanzee PBMCs treated with IFNs (165, 167, 171). It is therefore possible that SUN2 could exert anti-HIV activity if induced in other species, although this remains to be tested. When overexpressed in human cells, SUN2 exerted strain-specific antiviral activity as T/F HIV-1 viruses were less susceptible (167). Infection was blocked after DNA synthesis, before or at the point of nuclear entry, and was associated with drastic changes in nuclear morphology resulting from SUN2 overexpression. It is not clear why evidently global effects on nuclear morphology, should specifically inhibit certain HIV-1 strains and not others. Serial passage of HIV-1 in the presence of overexpressed SUN2 resulted in resistant viruses, largely conferred by the single capsid mutation P207S (167). The host cell cofactor CypA, that is recruited to incoming virions, was also implicated in targeting the capsid to SUN2 restriction, as CypA inhibitors partially relieved the block to infection in the presence of overexpressed SUN2, consistent with the notion that capsid–CypA interactions guide virion nuclear import pathways (37, 172).

While silencing or depletion of SUN2 in cell lines has been shown to have either no impact or very modest impact on HIV-1 infection (167, 170), silencing in primary T cells inhibited infection and produced a large defect in replication assays, leading the authors to surmise that SUN2 acts as a cofactor for HIV-1 (168). This was again proposed to be dependent on CypA recruitment to the capsid, as CypA inhibitors had no additive effects with SUN2 silencing (168). However, the defect in primary T cells has since been convincingly attributed to defects in T cell proliferation, activation status and viability resulting from SUN2 silencing (169). Discrepancies in cell viability between the two studies could be explained by depletion efficiencies and the duration of silencing experiments. In summary, endogenous SUN2 appears to play a central role in T cell proliferation and activation, which indirectly makes it essential for HIV-1 infection of activated primary T cells in culture. Due to difficulties in infecting resting primary CD4+ T cells *in vitro*, it will be difficult to establish whether SUN2 has additional cofactor roles in infection.

## MxB

MxA and MxB (Mx1 and Mx2 in mice) are ISGs that belong to the dynamin-like GTPase superfamily. Human MxA has broad antiviral activity against both RNA and DNA viruses, best characterized against influenza A viruses (173). By contrast, MxB has only been shown to have antiviral activity against certain retroviruses. Three different groups simultaneously reported that MxB is a potent inhibitor of HIV-1 and contributes to IFNα-induced anti-HIV-1 activity in a range of cell types (174–176). Nonetheless, type 1 IFNs typically suppress HIV-1 DNA synthesis, whereas MxB appears to act after HIV-1 has completed viral DNA synthesis. This suggests that MxB can act against HIV-1 but that in a typical type 1 IFN response, another, as yet unidentified factor(s) restricts HIV-1 before the MxB induced block. Consistent with this notion, some studies have shown that MxB knock out does not reduce the antiviral activity of type 1 IFN against HIV-1 (177).

Human MxB is active against various HIV-1 strains, including different subtypes and T/F viruses (178). In comparison, HIV-2 and some SIV strains are less susceptible, and unrelated retroviruses including MLV, feline immunodeficiency virus, and equine infectious anemia virus appear resistant to the human protein (174). Divergent primate MxB orthologs have been shown to have different patterns of restriction indicating some degree of species specificity (179), although this is not as clearly defined as, for example, for TRIM5α. Differences in viral susceptibility map to the capsid protein, suggesting it is the target of MxB antiviral activity. MxB resistant capsid mutants have been identified in naturally occurring primary isolates (180). The fact that MxB resistance mutations exist naturally, but are not universal, suggest uneven or incomplete selection pressure on HIV-1 from MxB, consistent with it having a minor role in the IFN response against HIV-1.

Most studies have reported that MxB expression inhibits nuclear entry, evidenced by a reduction in 2-long terminal repeat (2-LTR) circles, which are likely only formed in the nucleus by the uniquely nuclear non-homologous end joining pathway (181). A subsequent defect, implying a second block, can also be observed in the level of integrated proviral DNA (174, 175). Liu et al. reported a defect in integration, but not nuclear entry (176). These discrepancies prompted a thorough investigation by Busnadiego et al. who showed that MxB expression reduced 2-LTR circles, but that this defect did not fully account for the greater defect observed in infectivity (179). They suggested that MxB may therefore inhibit subsequent stages of infection in the nucleus. While they concluded that integrase activity was unaffected, MxB expression significantly altered the distribution of integrated proviral DNA away from gene-dense regions, although it is not clear if this also accounted for the remaining defect in infectivity. Similar effects on integration targeting have been observed for capsid cofactor binding mutants that are thought to have altered nuclear import pathways (37). Interestingly, the genomic position of integrated proviruses has recently been linked to differences in proviral expression and latency (182), although no study has yet demonstrated how retargeted integration by MxB may impact infectivity or replication in spreading infections. We speculate that the restriction activity of factors like MxB could have a greater impact on HIV-1 infection *in vivo* by retargeting integration, the full consequences of which may not be apparent in single round HIV-GFP infection assays in cultured cells.

The antiviral activity of MxB appears dependent on HIV-1 cofactors, including CypA, which are recruited to the incoming capsids. Like naturally occurring resistance, MxB resistance mutations, generated by repeat passage of HIV-1 in the presence of MxB, were found to map to the capsid, for example, to the CypA binding loop residue A88 (176). We note that HIV-1 CA A88 is very conserved in HIV-1 M isolates (183). RNAi mediated silencing of CypA and chemical inhibition of capsid–CypA interactions with cyclosporine rescue the MxB-mediated block to infection, consistent with a role for CypA (184). The N74D capsid mutant, which cannot bind the cytoplasmic cofactor CSPF6, or nuclear pore component Nup153, is also less susceptible to inhibition by MxB (175). Current thinking is that recruitment of cofactors to the incoming virion targets it into a pathway where it may encounter MxB in the context of an IFNα response, potentially at the cytoplasmic face of the nuclear pore where MxB is localized (185). Based solely on *in vitro* binding assays, the cofactors are not thought to be required for binding of MxB to the capsid as MxB-capsid interactions are not affected by cofactor binding mutations (184, 186). However, whether HIV-1 cofactors have a role in recruitment of MxB to the capsid during infection has yet to be determined.

The capsid residues that are targeted by MxB have not yet been mapped. Sites associated with resistance, found throughout the capsid, are thought to affect capsid stability suggesting they might not be directly targeted (179, 180). *In vitro*-binding assays suggest that MxB can only interact with capsid hexamers, rather than monomers, suggesting avidity effects and leading to suggestions that MxB may recognize hexameric capsid patterns (186).

The mechanistic details of MxB antiviral activity are therefore not yet fully understood. In trying to gain insight into the mechanism, numerous studies have probed the importance of each MxB domain through comparisons to MxA and structure-guided mutagenesis. Like MxA, MxB has a GTPase domain, which is linked by a bundle signal element (BSE) to a carboxy terminal stalk domain (186). Surprisingly, and unlike MxA, neither the GTPase activity nor conformational communication through the BSE is required for MxB antiviral activity (175, 186, 187). MxB has an extended N terminal domain, not present in MxA, which is essential for *in vitro* binding to the capsid and antiviral activity (175, 179, 184, 187). Transfer of the human MxB N terminal domain (25 amino acids) onto canine MxB orthologs, and unrelated proteins, confers anti-HIV-1 activity, providing the chimeric protein is able to dimerize (188). This is consistent with structure-guided mutagenesis studies that have confirmed that MxB dimer or trimer formation, mediated by residues in the stalk domain, is required for anti-HIV-1 activity by increasing the avidity of MxB–capsid interactions (184, 189), much like TRIM5. A triple arginine motif in the N terminal domain has been suggested to directly bind to the capsid. This sequence is essential for restriction and introduction of the motif into non-restrictive MxB orthologs confers anti-HIV-1 activity (190).

The N terminal domain of MxB also contains a nuclear localization sequence (NLS), and MxB is able to shuttle between the nucleus and cytoplasm (191). Early studies with N-terminal truncation mutants that were unable to restrict HIV-1 led to conclusions that MxB nuclear localization is essential for activity (188). However, it is now thought that these studies were confounded by deletion of the MxB N-terminal capsid-binding motif. To deconvolute the two functions of the MxB N-terminus, a recent study made point mutations in the NLS, which did not compromise HIV-1 restriction, but prevented nuclear rim localization (188). This study also used leptinomycin B to prevent MxB nuclear export leading to an accumulation in the nucleus. This did not disrupt HIV-1 restriction. However, it is possible that residual cytoplasmic MxB was able to inhibit infection in these experiments and further studies are required to clarify these apparently contradictory reports and determine exactly where in the cell MxB restriction of HIV-1 takes place.

The N-terminal domain of MxB has been shown to be under diversifying positive selection in primates, consistent with a role in directly binding pathogens and with pathogen-driven evolution (192). However, the four amino acids found to be under positive selection did not include the triple arginine motif, or the NLS implicated in HIV-1 restriction. This suggests that MxB evolution may have been driven by other viral pathogens, implying broader antiviral activity (192). An alternative explanation is that we do not yet fully understand the interactions and mechanisms of inhibition of MxB against different lentiviruses. The N-terminal residues under selection were targeted by alanine scanning mutagenesis in a separate study with no apparent effect on HIV-1 inhibition (190). However, making evolutionary analysis-guided changes in MxB, rather than simply mutations to alanine, and testing antiviral specificity, may prove more informative.

An outstanding question regards the fate of MxB-restricted capsids in the infected cell. It has been proposed that MxB binding prevents uncoating, as accumulation of p24 capsid proteins has been observed with MxB expression (184). This was also based on indirect biochemical measurements using a “fate of capsid” assay, which compares the amount of “intact” viral cores that can be pelleted from infected cells in different conditions (193). Measuring uncoating in cells remains challenging and somewhat controversial, due to the rarity of infectious events and the possibility that the majority of events measured biochemically do not represent those leading to infection. Nonetheless, these experiments can be informative and understanding the effect of MxB on viral capsids as a whole is certainly worth pursuing.

## Schlafen (SLFN) 11

Schlafen genes are unique to mammalian cells; there are six human SLFN genes and all possess motifs shared with nucleic acid sensors RIG-I and MDA-5 (194). SLFN11 was originally suggested to restrict HIV-1 replication at the level of protein translation in human cell lines and activated primary CD4+ T cells (195). They suggest that SLFN11 counteracts HIV-1-induced changes in tRNA composition, which is presumed to relate to initiation of viral protein synthesis. These authors proposed that SLFN11 may exploit differential codon usage between viral and host proteins: lentiviral genomes have high frequencies of A nucleotides and favor rare codon usage, relative to host cells, with A/U in the third position. Thus, SLFN11 may exploit viral codon preferences to specifically attenuate viral protein synthesis. Electrophoretic mobility shift assays implied that SLFN11 might achieve this by binding and sequestering tRNA on which HIV-1 is dependent (195). More recent evidence suggests that overexpression of SLFN11 in HEK 293T cells reduces all protein production, including host protein translation in the absence of infection, with a bias toward genes that have not been codon optimized for the relevant host cell (196). SLFN11 gene expression is IFN induced and it may be more appropriate to consider SLFN11 as a broad-acting ISG that contributes to the antiviral state by mediating host cell translational shut-off, rather than a restriction factor specific to any particular virus or virus family (195, 197). The other human SLFN paralogs remain to be explored in this context.

## Immune Evasion Strategies of HIV

In comparison to large DNA viruses, such as herpes or pox viruses, which carry an armory of proteins capable of disabling all branches of the innate immune response, HIV-1 travels light, with just nine viral genes. The HIV accessory proteins, which are dedicated to antagonizing host defenses, are multifunctional and able to manipulate activity or expression of many target proteins (198–200). However, without the genetic capacity to initiate a global shutdown of host responses, evasion of detection is thought to be important for HIV-1 replication and particularly for transmission. As such HIV-1 has evolved a stealth strategy that operates throughout its lifecycle.

## Evasion of Nucleic Acid Innate Immune Sensing by the HIV-1 Capsid

Studies from our lab and others have demonstrated that HIV-1 infection is silent in MDM and does not activate NF-κB or IRF3 signaling, or a type I IFN response, if the viral prep is purified from inflammatory cytokines made by the viral producer cells (16, 33). This stealthy replication is in part dependent on the cytoplasmic exonuclease TREX1, which degrades HIV-1 reverse transcripts that would otherwise be sensed by cGAS leading to a type I IFN response (33, 42). In this way, HIV-1 exploits the negative regulatory role of TREX1 in modulating innate immune activation, which may have evolved to prevent sensing of mobile endogenous retrovirus genomes (201). Genetic polymorphisms that inactivate TREX1 cause some cases of Aicardi–Goutieres syndrome (mentioned earlier), a serious autoinflammatory condition characterized by high systemic levels of IFN (202).

The HIV-1 capsid plays a central role in evasion of cytoplasmic DNA sensing in MDM. The capsid recruits the cellular cofactors CypA and CPSF6, which somehow cloak HIV-1 replication and prevent detection of newly synthesized viral DNA during transit across the cytoplasm (33). CypA is a highly abundant cytoplasmic protein with prolyl-peptide isomerase activity, whose function is not well understood despite having been implicated in a range of cellular processes including innate immune signaling (203). CPSF6 is involved in mRNA processing in the nucleus, but can also be found in the cytoplasm (204). Both CypA and CPSF6 target the virus to particular nuclear import cofactors and influence integration site selection (37, 205). Both cofactors are essential for HIV-1 replication in MDM, as capsid mutants that are unable to recruit either CypA (P90A) or CPSF6 (N74D) trigger a type I IFN response that completely suppresses infection (33). RNAi-mediated depletion of CPSF6, or pharmacological inhibition of CypA, has the same effect. Blockade of IFN signaling rescues infection in each case, confirming the importance of innate immune evasion for successful infections. Sensing of the CypA binding mutant (P90A) was dependent on viral DNA synthesis and resulted in production of cGAMP. Non-immunosuppressive derivatives of cyclosporine A, which block CypA–capsid interactions, also triggered a type I IFN response that suppressed infection, demonstrating the potential for therapeutic intervention to promote innate immune responses.

We do not yet fully understand the mechanisms by which cofactor recruitment helps to cloak the incoming capsid and prevent sensing of viral DNA. An attractive hypothesis is that sequential cofactor binding acts as a “satnav,” by regulating the coordinated processes of DNA synthesis and uncoating, ensuring they happen in the right intracellular location, and at the right time, to avoid detection. This hypothesis is supported by structural studies that revealed that pairs of cytoplasmic and nuclear cofactors, for example, CypA/Nup358 and CPFS6/Nup153, have overlapping binding sites on the surface of the capsid (39), suggesting that an exchange of cofactor binding may happen at the nuclear pore to control uncoating and protected DNA synthesis.

Structural analysis has also revealed that the CPSF6/Nup153 binding site spans multiple subunits within capsid hexamers, suggesting that interactions with Nup153 can only take place with intact capsid cores. Taken altogether, these studies have added to growing evidence that the capsid stays intact until it reaches the nuclear pore, contrary to dogma that proposes uncoating occurs soon after the capsid enters the cytoplasm. Encapsidated DNA synthesis would allow RT to occur within the safety of the core, shielded from cytoplasmic DNA sensors and from TREX1 degradation (40). Indeed, intact capsids have been observed docked at the nuclear pore by electron and light microscopy (36, 38).

For in-core RT to be possible, dNTPs must be able to enter intact cores to fuel DNA synthesis. Jacques et al. recently discovered that capsid hexamers form an electrostatic transporter that can transport dNTPs (40). They demonstrated that the channel, with its electrostatic core comprising a ring of positively charged arginines, allowed RT within intact cores *in vitro*. Mutation of the key arginine at position CA18 led to decreases in dNTP binding, RT, and infectivity. On the outside of the CA, the channel is opened and closed by a dynamic molecular iris formed by a beta-hairpin structure. The beta-hairpin exists in different conformations in X-ray structures, suggesting its acts as a lid to regulate the electrostatic channel. This could provide the virus with a means of controlling entry of dNTPs and DNA synthesis by CA binding cofactors. Of course, linked processes, such as uncoating, could also be controlled in this way. However, it remains to be defined as to whether and how the channel is regulated in the host cell cytoplasm. The contribution of the channel and beta-hairpin in encapsidated RT and the mechanisms of evasion of innate immune sensing also require further study.

## Antagonism of Innate Immunity by HIV-1 Accessory Proteins

### Viral Infectivity Factor (Vif)

HIV-1 Vif is essential for viral replication in CD4+ T cells and some T-cell lines (206, 207). Importantly, *in vivo* studies show that SIV lacking Vif is less infectious, with reduced pathogenicity (208). One of the reasons for this reduced infectivity is that Vif-deleted viruses are restricted by APOBEC3 proteins (150). The best characterized function of Vif is its ability to counteract the antiviral effects of APOBEC3 proteins by targeting them for degradation in infected cells. This prevents them from being packaged into nascent virions and circumvents their antiviral activity (209). To do so, Vif hijacks the Cullin5 (Cul5) E3 ubiquitin ligase complex by mimicking its cellular substrate recognition subunit, SOCS2 (210). As such, it links A3s to the Cullin5 E3 ubiquitin ligase complex containing elonginB, elonginC, and Rbx-2 for polyubiquitination and subsequent degradation by the proteasome. Structural studies have revealed that interactions of Vif with different A3 proteins are mediated by its N terminus, whereas the C-terminus recruits the Cul5 E3 ubiquitin ligase complex proteins (211). In crystal structures, Vif adopts an elongated cone-like shape, with two domains surrounding the zinc-binding region, when bound to the Cul5 E3 ligase complex (212). The zinc-binding region stabilizes Vif structure by coordinating zinc through an HCCH motif. Vif uses three distinct regions in its N terminus to bind A3 proteins, which affords it broad specificity. The ^14^DRMR^17^ motif is used to interact with A3F, A3C, and A3D (213, 214). The ^40^YRHHY^44^ motif is used to interact with A3G (215) and residues ^39^F and ^48^H are used to interact with A3H (216).

Although proteasomal degradation is the primary mechanism by which Vif antagonizes A3G, there is evidence that Vif can also decrease translation of A3G mRNA (217), prevent A3G packaging into virions (218), and inhibit cytidine deamination activity of A3G (219). Various strategies used by Vif certainly hinder A3G packaging into virions; however, low levels of enzymatically active A3G can be detected in wild-type HIV-1 virions (220), resulting in sublethal deamination of the viral DNA (221). Several lines of research convincingly show that non-catastrophic increases in HIV-1 mutation rates, induced by low level A3G expression, may be beneficial for the virus and allow, for example, generation of antiretroviral resistance (222), escape from cytotoxic T lymphocytes (223) and co-receptor switching (224, 225).

Like other lentiviral accessory proteins, interaction of Vif with A3 proteins is species specific and is thought to present a cross-species transmission barrier. HIV-1 Vif degrades human but not simian A3G. Specificity can be determined by a single residue, for example, at position 128 of A3G, which dictates binding of Vif and therefore species-specific A3G antagonism (224). Species specificity of antagonism of A3G by Vif is indicative of the arms race between pathogens and their hosts, resulting in continuous selection pressure that drives evolution of this protein (226, 227).

Additional functions for Vif have recently been proposed by proteomic studies seeking additional targets for Vif degradation. Greenwood et al. identified host cell protein PP2A, which belongs to the B56 family of serine/threonine phosphatases involved in numerous cellular processes, as a novel Vif target (199, 228). By studying changes in the proteome of an HIV-1 infected T-cell line, they found that PP2A had the same pattern of temporal loss as APOBEC3 proteins suggesting PP2A as a Vif target. Subsequently, the authors confirmed that, indeed, Vif targets all five members of the B56 family for Cul5-dependent proteasomal degradation. In contrast to APOBEC3 antagonism by Vif, targeting of PP2A was found to be a conserved function of lentiviral Vif proteins as Vif proteins from different primate and non-primate lineages could target human PP2A. Currently, it is unclear why Vif targets the PP2A complex.

### Vpr

Vpr, an accessory protein of around 96 amino acids, is packaged into viral particles *via* interactions with Gag derived p6 (229). Virion incorporation suggests it functions during viral entry or egress from infected cells. Although present in all primate lentiviruses, its sequence is highly variable between viruses and even within species. Numerous functions have been proposed for Vpr (230); however, its role in HIV-1 infection has remained poorly defined and its function remains enigmatic. This is partly because, while Vpr is typically dispensable for replication in cultured CD4+ T cells, there are reports of Vpr-dependent HIV-1 replication in MDMs (231), suggesting that its function might only be apparent under certain conditions. Here, we discuss only the proposed functions of Vpr relating to innate immunity.

Various studies have shown that Vpr modulates innate immune activation by regulating activation of transcription factors, IRF3 and NF-κB, during early stages of the HIV-1 life cycle. In TZM-bl cells reconstituted with STING, Vpr was found to inhibit sensing of HIV-1 by blocking translocation of IRF3 into the nucleus (232). On the other hand, in PBMCs, and the Jurkat T-cell line, Vpr was found to degrade IRF3 (233). In contrast to the effects of Vpr on IRF3, NF-κB has been described to be activated by Vpr, potentiating innate sensing of HIV-1 in CD4+ T cells and DCs (62, 234).

Like the Vpr related protein Vpx, Vpr usurps the Cul4-DCAF1 E3 ubiquitin ligase complex to target proteins for proteasomal degradation (235). The most extensively studied function of Vpr is to cause cell-cycle arrest at the G2 to mitosis (G2/M) transition. A 2014 study showed how Vpr can manipulate an endonuclease complex to arrest cell cycle and proposed that this prevents innate immune sensing of the viral DNA (236). The data suggested that Vpr interacts directly with SLX4, which is implicated in DNA damage repair pathways. SLX4 recruits structure-specific endonucleases (SSEs) MUS81-EME1, ERCC1–ERCC4, and SLX1 to form a complex (SLX4com) that repairs DNA damage. The activity of SSEs is kept under tight control during cell cycle. They are only activated at the G2/M transition, for example, by kinases such as polo-like kinase 1 (PLK1) leading to resolution of stalled replication forks and maintenance of genomic integrity (237). Laguette et al. proposed that Vpr recruits PLK1 to the SLX4com before the G2/M transition. PLK1 then prematurely activates SLX4com by phosphorylating EME1 resulting in abnormal processing of replication forks that eventually leads to replication stress and cell-cycle arrest at the G2/M transition. This function of Vpr is dependent on Cul4-DCAF1 ubiquitin E3 ligase complex as the DCAF1 binding mutant, VprQ65R, is unable to cause cell-cycle arrest. Furthermore, SLX4 was found to bind HIV-1 reverse transcripts only in the presence of Vpr suggesting that Vpr may recruit SLX4 to process HIV-1 reverse transcripts and prevent innate sensing.

These findings raise important questions of how Vpr manipulates the SLX4 complex to degrade viral DNA and evade innate sensing without suppressing productive infection. Importantly, the significance of SLX4 activation by Vpr during HIV-1 replication was not demonstrated in this study. The relevance of the Vpr interaction with SLX4 is undermined by the recent suggestion that Vpr from certain HIV-1 isolates are unable to interact with SLX4 (238). However, species-specific Vpr–SLX4 interactions support the importance of this interaction. SIV Vpr proteins from African green monkeys that do not arrest cell cycle in human cells can interact with the SLX4com in African green monkey cells and cause cell-cycle arrest (239). The role of SLX4 in HIV-1 replication and Vpr activity certainly warrants further investigation. It is also likely that Vpr targets, as yet unidentified, factors and pathways as evidenced by recent proteomic screens identifying putative Vpr targets (200, 240). A major challenge is to identify a reliable assay for Vpr function and a corresponding replication assay that links target degradation to viral replication as was the key to understanding the relationships between HIV-1 Vif and APOBECs, Vpu/Nef and Tetherin, and Nef and SERINC3/5 (241, 242).

### Vpu

A detailed description of the roles of Vpu as an antagonist of the restriction factor tetherin, which prevents viral budding and release from infected cells, and in the regulation of host transmembrane proteins, are described elsewhere in this edition (Neil lab review). Its anti-tetherin activity also implicates Vpu in innate intracellular signaling pathways, because tetherin also acts as a PRR that activates signaling cascades upon recruitment of HIV virions (84, 85). Intriguingly, another study examining tetherin signaling demonstrated that tetherin has a long and a short isoform, that are, as for MAVS, derived from alternate start codons (243, 244). Also like MAVS, the long form can activate an innate immune signal whereas the short form cannot. Intriguingly, Vpu preferentially targets the long signaling form of tetherin, despite the fact that the short form is competent for tethering newly formed virions (243). In the light of these data, we consider that the name tetherin is rather misleading, in fact, being tethered may not be disadvantageous to HIV-1, particularly given this feature aids cell-to-cell spread in T cells (245). Indeed, preferential targeting of the long form of tetherin by HIV-1 suggests that it is signaling and its consequences that exert the dominant pressure on the virus. This is consistent with a model in which the most important feature of restriction factors is their PRR function, which can protect many cells through initiating IFN responses, rather than their restriction function, which is focused on individual viral particles. Having said this, tetherin signaling may be a recent adaptation given that simian tetherin variants were found to be unable to activate signaling when expressed in 293T cells (84). Of course, these proteins may signal in their cognate species and the role of signaling in viral restriction by tetherin requires further study. Furthermore, cell-free virus is required for transmission and therefore antagonism of tetherin by Vpu is critical. Tethered viruses may also enhance antibody-dependent cell-mediated cytotoxicity (246).

In contrast to other accessory proteins, Vpu is exclusively encoded by HIV-1 lineage viruses and is absent from HIV-2 clades. It is not packaged inside the virion, and its Rev-dependent expression occurs late in the viral lifecycle (49). Vpu potently inhibits NF-κB activation and ensuing ISG expression; this likely requires viral integration in primary myeloid and CD4+ T cells (62, 247, 248). As mentioned earlier, activation of NF-κB occurs downstream of multiple innate sensing pathways to drive antiviral gene expression. Paradoxically, NF-κB activation is also implicated in driving HIV-1 and HIV-2 proviral transcription (249). Thus, primate lentiviruses may encode factors to closely regulate NF-κB activation at different stages in the lifecycle to strike a balance between shutting down antiviral responses and augmenting viral gene expression. In particular Nef and Tat, expressed at high levels early in the viral lifecycle, have been shown to increase virus replication by promoting NF-κB activation (250, 251). Intriguingly, Vpu’s role as an antagonist of innate immune signaling is independent of tetherin antagonism and is apparently conserved between all lineages of SIV and HIV-1 containing Vpu (except HIV-1 group N) (62, 248). Several reports show that Vpu disrupts NF-κB activation downstream of a range of exogenous and overexpression stimuli that are not specifically related to innate signaling (62, 84, 85, 248). Besides NF-κB, there are also conflicting data regarding antagonism of IRF3 by Vpu (248, 252, 253). It seems likely that host cell type and activation status may significantly impact the role of Vpu in the context of intracellular innate immune responses to HIV, which has yet to be fully elucidated.

## Future Perspectives

The last decade has seen an extraordinary expansion in our understanding of HIV and its interaction with the cell-autonomous innate immune system, especially pertaining to the field of DNA sensing. We are beginning to understand the complexities of the cellular responses to HIV-1 and the subtleties of HIV evasion strategies in different cell types. Viruses are the masters of compromise, able to switch roles between viral accessory proteins or finely tune their behavior with as little as single amino acid changes. In our view, particularly pertinent studies are those that explain the differences in cofactor requirements or innate evasion strategies in cell lines versus primary cells. Of course, cell lines make tractable models for HIV infection, but we must remember that, in many cases, this is because of defects in cell-autonomous innate immunity related to their cancer origins, and so we must be cautious in interpreting experiments studying tropism in cells that cannot mount authentic responses. Also important are studies that take into account the fact that cells communicate through cytokine and cGAMP secretion (63, 72). Humanized mouse models are now allowing sophisticated and relatively cheap *in vivo* investigation of HIV therapeutics and innate responses (254–256). Similar studies considering innate immunity may eventually be more informative for HIV-1 than simian models given that simian lentiviruses, such as the Vpx encoding SIVmac, have different cellular tropisms and likely innate immune relationships with their hosts. Mouse models may be particularly effective for the study of HIV-1 tropism in human T cells *in vivo*, which must be activated for HIV-1 replication *in vitro*, thereby masking innate responses and cytokine secretion profiles in response to the virus itself.

It is clear that HIV adaptation to host defenses influences the HIV-1 lifecycle at every stage. As our understanding of HIV-1 innate immune evasion increases, comparative studies between the different SIV and HIV strains are becoming increasingly informative and may shed light on the determinants of pandemicity. Manipulation of these critical host–virus interactions offers tantalizing opportunities for development of novel therapies. However, excitement at the prospect of translation of our rapidly expanding knowledge base must be tempered by the contradictions and uncertainties in the field; there is still much to be understood. The literature tells us that there is great diversity in the innate immune capacities of each cell type relevant to HIV infection. One major and evolving challenge is understanding the dynamic relationship between intracellular immunity and the specific circumstances of each individual cell or cell population: cell location, cell-cycle status, relative cytokine exposure, for example, will variegate each cell’s interaction with HIV. We view HIV as the ultimate tool for molecular cell biology, which, correctly deployed, will teach us a great deal of fundamental human biology and continue to transform our understanding of health and disease leading to innovative new tools and therapeutics.

## Author Contributions

All authors contributed to conception, drafting, and final approval and are accountable for the integrity of the final manuscript. RS and LT contributed equally to this article.

## Conflict of Interest Statement

The authors declare that the research was conducted in the absence of any commercial or financial relationships that could be construed as a potential conflict of interest.
